# Continuous Improvement of Digital Health Applications Linked to Real-World Performance Monitoring: Safe Moving Targets?

**DOI:** 10.1016/j.mcpdig.2023.05.010

**Published:** 2023-06-25

**Authors:** Stephen Gilbert, Andreia Pimenta, Ashley Stratton-Powell, Cindy Welzel, Tom Melvin

**Affiliations:** a Else Kröner Fresenius Center for Digital Health, Technische Universität Dresden, Dresden, Germany; b Ada Health GmbH, Berlin, Germany; c RQM+, Blenheim Court, Nottingham, United Kingdom; d School of Medicine, Trinity College, University of Dublin, Dublin, Ireland

## Abstract

Real-time high-quality data on the performance of digital health applications is needed for feedback-led optimization and to ensure safety and performance, particularly if they will have on-market updates. Developers must verify that applications accurately and consistently fulfill their intended purpose in real-world use. In particular, new thinking from regulators recognizes the importance of monitoring real-world performance. It is acknowledged that real-world data can deliver information from wider patient populations than are generally included in controlled studies, and in certain circumstances, this can enable extensions of the application’s intended purpose. Proactive postmarket surveillance surveys are an important source of real-world data that are distinct from clinical investigations but may vary in quality and, if inappropriately designed, can be subject to uncontrolled bias. We aimed to describe the practice of real-world data gathering through patient-reported and clinician-reported outcomes and high-quality surveys and identify challenges, uncertainties, and health policy gaps.

## Search strategy and selection criteria

References for this Health Policy Manuscript were identified through searches of PubMed with the following search terms: *digital health*; *post market*, *surveillance*, *real-world {evidence/data/performance monitoring}*; from 2017 until December 2022. The articles were also identified through searches of the authors’ own files and through relevant citations in the identified literature. Only articles published in English were reviewed. The final reference list was generated on the basis of the originality and relevance to the broad scope of this review.

**Article Highlights**

• Real-world performance monitoring is important to gather real-time high-quality data on the performance of digital health applications and, particularly, important for adaptive tools
• Real-world data deliver information from wider patient populations, which can enable the extension of the application’s intended purpose
• Proactive postmarket surveillance is an important source of real-world data but may vary in quality and can be subject to uncontrolled bias
• Patient-reported and clinician-reported outcomes and high-quality surveys are important approaches for real-world data gathering for digital health applications

**Figure 1:**
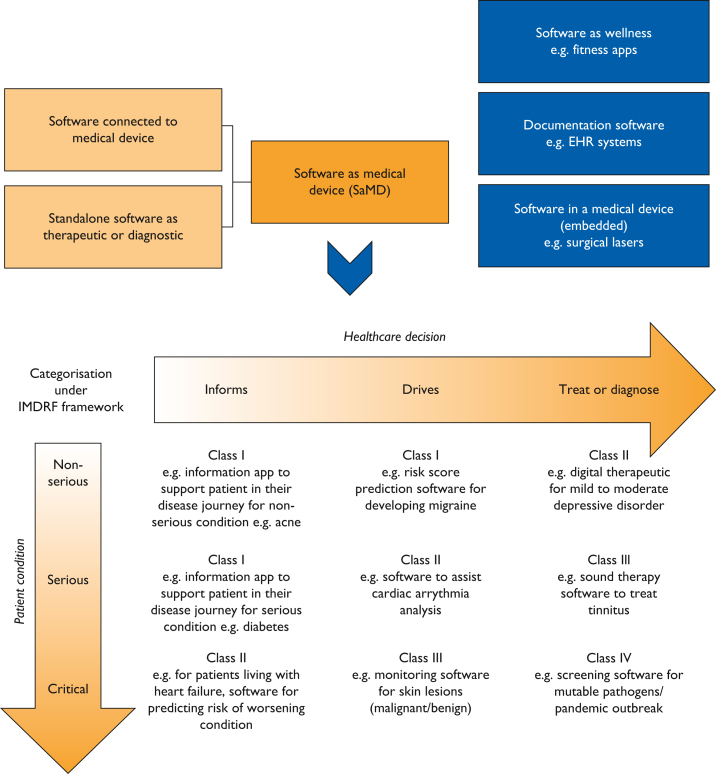
Overview of software in health care and risk categories of Software as Medical Device (SaMD) under the international IMDRF framework,^3^ which is reflected in the regulations of most countries. IMDRF, International Medical Device Regulators Forum.

Medical device software is an important part of modern and integrated health care and is increasingly being used by clinicians, patients, and health care institutions, affecting treatment decisions.^1^^,^^2^ National regulations have different classification schemes that determine what software use cases are considered for medical devices and in which risk classes they fall.^3^, ^4^, ^5^, ^6^, ^7^, ^8^, ^9^ Medical device software is divided into software as a medical device (SaMD) and software in a medical device. SaMD may drive critical diagnosis or treatment decisions, whereas software in a medical device may control active implants, such as implantable pacemakers.^3^^,^^4^^,^^10^ For these use cases, prospective clinical investigation (CI) data are generally needed before market approval (Figure 1).^11^ However, many SaMDs are of low or intermediate risk and take the form of mobile device digital health applications (DHAs)^5^^,^^12^^,^^13^ in the zone between safety-critical medical applications and wellness applications.^5^^,^^12^^,^^13^ Increasing numbers of DHAs are being made available to consumers through application stores, many of which are medical devices, requiring regulatory approval.^14^, ^15^, ^16^ It was recognized that there have been insufficient oversight procedures for what constitutes regulated SaMD on the application stores,^15^ and there has been a related reclassification of many SaMD applications in the European Union (EU) into a higher risk category.^17^ Simultaneously, the application of traditional regulatory approaches to lower-risk and intermediate-risk SaMDs may not be the only approach to ensuring the safety and performance of these devices, and there is increasing acknowledgment that, as technologies evolve, regulatory frameworks and approaches also need to evolve. We aimed to review the literature on the most recent thinking in governance and oversight of on-market adaptation and predetermined change plans for DHA technologies, considering alongside the needs and requirements for clinical evidence and approvals of changes needed under current regulatory frameworks.

## On-Market Adaptive DHAs and Artificial Intelligence–Based or Machine Learning–Based SaMD

**Figure 2:**
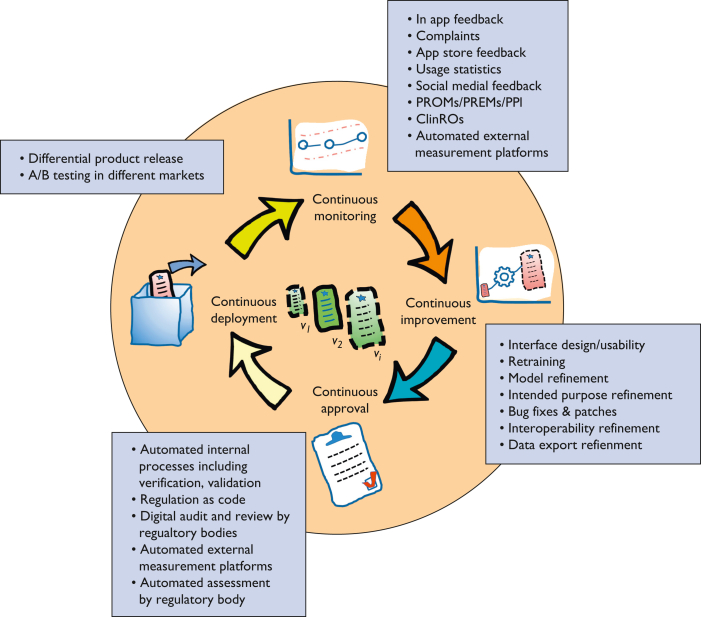
The concept of continuous monitoring, continuous improvement, continuous approval, and continuous release of DHAs, which has been introduced in recent thinking from regulatory bodies.^19^^,^^23^

To a much greater degree than for physical or hardware-based devices, change, or adaptation is a core aspect of the general philosophy of software development. DHAs are often also artificial intelligence (AI) or machine learning (ML) based, and adaptation on the basis of learning is also a central principle of this technology.^18^^,^^19^ Current regulatory approaches make it effectively impossible to have compliant real-time or near–real-time adaptive/continuous learning AI-based SaMD on the market (often referred to as adaptive AIs), owing to rigid change control frameworks.^18^, ^19^, ^20^, ^21^ This approach, under which both software and AI-prediction models are effectively fixed once in the market, may remain the central paradigm. It is important to recognize that it is not an evidence-based approach but is on the basis of an adoption of general medical device design conventions.^18^^,^^19^ However, there are proposed regulatory approaches in the United States and United Kingdom, and to a lesser extent the EU, which are acknowledging the role of adaptability in AI-based technologies and are looking toward enabling this through real-world performance (RWP)-monitoring approaches.^18^^,^^19^^,^^22^^,^^23^ The methods proposed include predetermined change control plans (PCCPs) and SaMD prespecifications. As has now been acknowledged by some regulators, iterative and incremental software change does not only apply to AI/ML methods, and DHAs are inherently amenable to adaptation in their design through human/developer feedback–based learning and update.^18^^,^^19^^,^^23^ Indeed, it could be considered an anomaly that initial frameworks for adaptive SaMD^22^ have considered ML-based adaptation as inherently a different category of change than incremental human developer changes, on the basis of feedback. The changes that result from human developers are not inherently higher risk than changes in a prediction model in the process of retraining on data, and may lower risk, having passed through change control and risk assessment processes. From first principles, human-led changes are no more or less likely to be safe than AI-based changes. Common incremental changes include but are not limited to updates relating to operating system updates, dependent software components and libraries, bug fixes, security updates, user interface/user experience changes, and routine updates for security.^24^^,^^25^ Current approaches to regulation accept some degree of iterative adaptive development, with the classification of nonsubstantial and substantial changes of software, the latter requiring advance approval by regulatory bodies, particularly for high-risk devices.^18^ Newly proposed approaches in the United Kingdom would go beyond this, by applying the PCCP concept, first developed in the context of changes because of ML, to changes coded by human software developers.^19^^,^^23^ The degree to which this would extend current regulatory approaches is not certain. It is also uncertain the degree that PCCPs would enable continuous delivery, let alone continuous deployment, concepts shown in Figure 2. The application of this concept to SaMD is controversial, and it remains to be seen the degree to which it will actually be introduced into regulatory approaches. When PCCP-based approaches are introduced, either for general DHAs or particularly for AI/ML-based SaMD, this will require a transformative increase of both the focus on and the need for postmarket surveillance (PMS)/real-world data (RWD) methods, and this will require a range of RWP-monitoring approaches and tools suited for different use cases and evidence needs.

### RWP Monitoring of DHAs and AI/ML-Based SaMD

For all medical devices, such as SaMD, the introduction of the Medical Device Regulation (EU) 745/2017 formalized the requirement for manufacturers to continually and proactively demonstrate the safety and performance after their market approval, in a process known as postmarket clinical follow-up (PMCF).^11^^,^^26^ The approaches of all these countries recognize the importance of surveillance and RWD, such as registries, electronic health record data, and surveys, as forms of clinical data for lower-risk DHAs.^27^, ^28^, ^29^, ^30^, ^31^, ^32^ Indeed, in the context of lower-risk and intermediate-risk class DHAs, RWP monitoring, a form of PMS, can replace the need for formal postmarket CIs (Figure 1).^33^ However, there is the requirement for highly structured and certified processes for development, assessment, release, and surveillance.^34^^,^^35^ In addition, there is the requirement for sufficient premarket clinical data that demonstrate safety and benefit,^36^ and this can be provided through peer-reviewed reports and available postmarket data, provided that the developer can establish that this is adequate. Nonetheless, CIs may be necessary to provide data that are not available through other sources, to establish clinical validation, and to answer specific safety or performance questions.^37^

Real-world data are also relevant to the lifting of devices from EU certification under legacy regulations to the updated regulatory approvals, in the so-called transition period for older DHAs, between 2021 and 2028.^11^^,^^38^ In this case, RWD is particularly applicable because the typical risk profile of these products is likely to be known and considered to be acceptable. The task of identifying risks arising from misuse and off-label use can be more effectively identified through the less-targeted and broader data collection approaches, which are characteristic of RWP monitoring.

### Purpose of RWP Monitoring

Proactive PMS (such as PMCF) is generally required for all devices, but a tailored approach can be adopted, depending on the factors listed (Table 1).^39^, ^40^, ^41^ For some DHAs, minimal PMCF can be justified, which may include the scanning of journal articles. Whether proactive activities are required or not will depend on the product‘s intended purpose, the approach to its postapproval development and release, and the level of novelty. Moreover, clinical evidence gaps at the time of approval affect the degree of clinical evidence required: for example, if the clinical data did not capture all possible target populations, clinical indications, use scenarios, or claims comprehensively.^32^ Active PMS/RWP monitoring may allow the identification of important unknown data on the device (Table 1).^42^

**Table 1:** Important Unknown Data on the Device That Can Be Identified Through PMS/RWP Monitoring

Aspect	Regulatory Relevance of New Data	References
1	Potential new applications of the device	39
2	Changes to the degree of benefit of the use of the device over time	39
3	Differential effect on subgroups of the populations using it	40
4	Side-effects that are different than anticipated, qualitatively or quantitatively	41

PMS, postmarket surveillance; RWP, real-world performance.

An additional driver for performing proactive PMS activities can be used to deliver direct or supportive data on the cost-effectiveness of the SaMD/SiMD, which are rarely captured in the clinical evidence used for the initial market approval. For some but not all SaMDs, quality of life or other indicators of health economic performance may be relevant for Health Technology Assessments (HTAs), reimbursement, and tendering processes.^43^ This is relevant for SaMDs that will be integrated with or prescribed by major health care networks (eg, UK National Health Service) and for which the data generated for initial regulatory approval/conformity assessment is often not the same because the data required for scaling the product. Recent approaches to HTA in the United Kingdom have incorporated not only the consideration that the DHA may further develop after release but also looked to coordinate approval and HTA approaches.^44^, ^45^, ^46^ For consumer DHAs, which are often funded through consumer payments, similar data gathering may be relevant to pricing, to the further refinement and development of business models, for product optimization, and for forming new product hypotheses.

### Methods for RWP Monitoring Relevant to DHAs

Real-world data include analyses of electronic health records (such as demographic characteristics, anamnesis, family history, all clinical test results, and medication data), health insurance claims, user surveys, patient surveys, wearable and DHA-derived data (from DHAs other than the DHA under surveillance), environmental data, user location data, social media analytics, and web and literature data.^47^ Of these sources of data, 2 forms that are being increasingly applied and have particular importance are feedback from individuals in the target populations (often but not always patients) and feedback from individuals in the user population (patients, other lay-users, health care providers [HCPs]). Although these data sources may be more subjective than analysis-based or sensor-based data streams (eg, blood test data and environment sensor data), they have the advantage of providing highly nuanced data regarding the real-life experience of the individuals interacting with the devices—data that must be considered in the evaluation of DHAs. The outside-of-clinical-investigation surveying of user and target population perspectives are recognized as an important source of data for PMS in international frameworks.^48^ Although surveys are recognized as an important data source, literature and regulatory guidance also acknowledges that they can vary in quality, can be subject to bias, and have some limitations in their data applicability.^32^^,^^47^ However, regulations and associated guidance do not define surveys, and although they have come to prominence, there is little information on their design, approval, or conduct. The developments of regulatory thought and approaches for surveying user and target population opinion are an important health policy topic in digital health and of high relevance to HCPs, patients, payers, regulators, and DHA developers. Future approaches for RWP monitoring may include the use of depersonalized patient/citizen data relating to DHAs, obtained through application to national data access bodies, under the dataspace frameworks.^49^

### RWP Monitoring in Context of Application A/B-Test Experiments

Postmarket surveillance and RWD gathering are generally considered to be observational research, both in guidelines and in proposals of the monitoring of AI/ML-based SaMD through approaches for monitoring adaptive change.^22^ If change-monitoring approaches are extended to agile/continuous software development,^19^^,^^23^ then a logical extension of this would be that surveys would be not only used for observation of the performance of a single released version but also extended to the comparison of multiple versions released simultaneously. For conventional hardware devices, having different versions on the global market at the same time is unusual or temporary in practice. However, for digital technologies, it is more common in the evaluation of performance and linked to product development and obtaining feedback.^2^ If on-market adaptivity of SaMD DHAs is to be enabled, then it will be necessary to adopt novel methods for RWP monitoring. It may be necessary to borrow from methods that have been standardized for real-time network-based evaluation of application updates for over a decade^50^ but that have not been regarded as part of the validation and evaluation of SaMD. For non-SaMD applications, it is commonplace to differentially release changes to different regions, to specific subgroups of users, or with random sampling of users. The effects of the changes are then monitored. This can be performed through web-based randomized controlled experiments. There are a range of designs, for example, A/B tests, which can compare 2 system features. These tools are used to optimize web page design; however, they adopt a number of similar methods to traditional CI design, for example, regarding hypothesis testing or statistical rationales.^50^ There is no inherent reason that these test approaches could not be used for PMS/RWP monitoring, provided that they were conducted in an ethical, rigorous, and recorded manner. This would be assisted through formal guidelines on how to conduct these types of studies ethically. It is critical that these approaches are only applied to optimization and continued improvement and never expose the user to unacceptable risk, and the developer should specifically assess risk, ethical approval, and regulatory requirements before using them.

A recent development adopted in the premarket approval phase of DHA development has potential to be applied in the postmarket phase, possibly as a part of A/B testing. In this approach, 2-phase mixed-methods research is applied. An observational and adaptation phase is followed by a detailed evaluation phase.^51^, ^52^, ^53^ In the first phase, qualitative and quantitative user experience research is performed using structured interviews and surveys, assessing the target population and/or the HCP-user interaction with DHA and their perceptions of it. At the end of this phase, the DHA is modified on the basis of the feedback. In the second phase, definitive evidence is generated regarding the performance and clinical benefit of the DHA, potentially through a CI. Provided that the changes made to the DHA were within those specified in the PCCP, this approach could be applied to on-market products, in 2 phases as described, or in more iterative phases of insight—generation, adaptation, and evidence generation, with statistical comparison with previous DHA versions.

### The Use of Surveys for RWP Monitoring

Surveying users for information on postmarket safety and performance is a common practice for medical device developers^32^^,^^54^ and is highly applicable to DHAs. The approach is predominantly used to supplement preexisting clinical evidence, obtaining real-time information from those using the medical device is invaluable to achieving a responsive RWP-monitoring system. This approach is particularly important to the evidence-generation approval of SaMDs because user feedback loops are an inherent part of software and user interface development approaches and because surveys can be efficiently delivered from within the DHAs themselves.^32^^,^^54^ However, there is no formal definition of a survey in the context of PMS or RWP monitoring, in regulations, associated guidelines, or harmonized normative standards. Internationally, the ethical and legal requirements and frameworks for surveys are often unclear. This is in contrast to CIs, which have strict approval, monitoring, and oversight procedures that are highly regulated, and although requirements vary internationally, these are always clearly specified and defined.

Among the developers of DHAs, there is often a lack of certainty of the borderline between activities that could fall under the definition of RWD data collection as part of proactive PMS activities and a second group of activities that meet the definition of a CI, such as under Medical Device Regulation (EU) 745/2017 and in associated normative standards^11^^,^^55^ and under the Code of Federal Regulations Title 21.^56^ Survey developers often face a catch-22 situation; although regulations include surveys as an approach for PMS, there is the implication that they are more flexible and are less demanding than CIs, but when regulatory bodies assess medical device quality systems and the evidence for device approval, there is often pressure for procedures to be systematized in a way that resembles CIs.^57^ Although some survey approaches may take place within fully digitized CI registries, which would have highly determined approaches, with monitoring according to Good Clinical Practice, and consideration of an ethical approval committee, it is also clear that surveys will have to be used in more flexible frameworks, if their role in the RWP monitoring of AI/ML and continuous development DHAs is to be realized. These surveys may be more informal and may resemble in-application feedback surveys and may in some scenarios be without specific ethical approval or formal monitoring. It is likely that there will be a continuum of approaches between these extremes, and future regulatory policy will need to clarify the types of approval processes required and the types of purpose for which data can be applied.^32^^,^^47^^,^^58^

### Survey Instruments in RWP Monitoring

Data for RWP monitoring can be gathered, at least partly, from patient-generated evidence measures, such as patient-reported outcome measures (PROMs) (including patient quality-of-life values), patient preference information, and patient-reported experience measures.^59^ Patient-reported outcome measures are patient-centered tools that can be used for the direct reporting of health outcomes by the target population of the DHA. A recent review identified 315 generic and condition specific validated PROMs, with the highest number for musculoskeletal conditions, cancer, and gastrointestinal conditions.^60^ Another important source of information is HCP/clinician-generated evidence measures, such as clinician-reported outcomes (ClinROs, also known as CROMs).^61^ These are validated outcome measures for use by the HCPs for evaluation of interventions or other clinical scenarios, and they focus on assessments of how patients feel, function, or survive.^60^^,^^61^

Relevant information for RWP monitoring is not limited to formal and prevalidated instruments for clinical outcome assessments alone, and wider measurement of the effect of a digital tool may be relevant to its regulatory, health economic, HTA, and product assessment (eg, usability issues, patient preferences, interoperability and data sharing, data privacy, and security).^30^^,^^62^ The use of off-the-shelf–validated surveys in PMS/RWE is contingent on the specific surveillance needs and clinical evidence gaps of the DHA. If an appropriate off-the-shelf instrument cannot be identified, then the development of a device or scenario specific survey will be required. This will often be guided by features of existing validated instruments but may require validation if substantially new.

Although traditionally focused on quantitative analyses, regulatory approvals/conformity assessment, and HTAs can be enriched and guided by qualitative data collection—for example, usability engineering approaches generally include both observational and qualitative assessment of user perspectives alongside validated quantitative survey instruments. The System Usability Scale^63^ can be used for the assessment of usability, and the Mobile App Rating Scale can be used for systematized assessment of a mobile application's quality, including in comparison with the state of the art.^64^ These concepts can be extended to other PROMs. Qualitative and mixed approaches may be useful for exploring patient experience in areas such as preferences, and acceptability (eg, “Do you have any feedback on the use of X product?”). Although surveys are not generally seen as an intervention, the conduct of a survey for the collection of clinical data are in some countries defined as research and requires ethical approval from designated ethics committees.^65^

### Quality in RWP-Monitoring Surveys for DHAs

Real-world performance monitoring aims to capture the overall use of the application (both intended and off-label use) in the real-world scenario of use, minimizing selection bias or influence of recorded data through rigid inclusion or exclusion criteria. Moreover, RWP-monitoring surveys should have approaches, evidenced in their design and reporting, which avoid bias and ensure rigor, as described in Table 2.^66^^,^^67^

**Table 2:** Quality of Design Criteria for RWP-Monitoring Surveys

Aspect	Quality of Design Criteria
1	Minimal or no inconvenience for users;
2	No effect of the survey on the users present or future interaction with the DHA
3	Sample users to ensure respondents are representative of real-world use population
4	Highly usable, eg, through inclusion of visual analog scale (VAS)^67^-like functionality
5	Thoroughly and appropriately validated for the specific use case, population, and scenario
6	Should record the users reason for use and route to access of the DHA
7	Complete demographic information on participants available
8	Complete medical information on participants available
9	Real-time surveying, analysis, and visualization: ie, a data-dashboard approach
10	For some use cases: prompted questions on the basis of specific use scenarios

DHA, digital health application; RWP, real-world performance.

**Figure 3:**
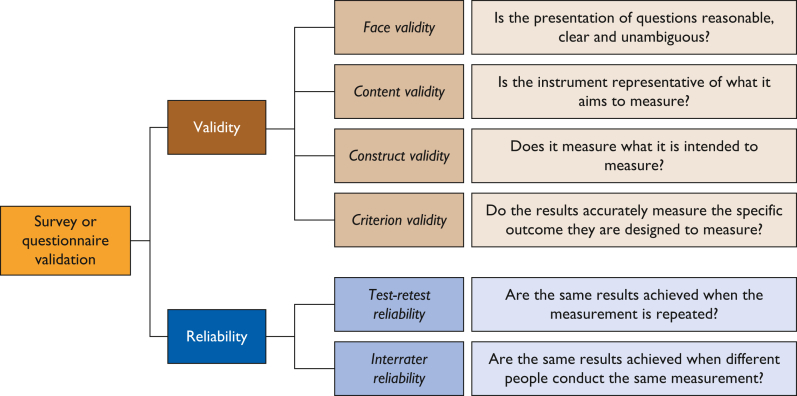
Aspects to consider in survey validation.

Survey validation is the process of ensuring that the survey accurately measures what it is intended to measure and that the data collected from the survey is reliable and valid. Methods for survey validation include testing the survey with a small group of people to identify any potential issues and making changes to the survey on the basis of that feedback (Figure 3).^68^^,^^69^

Given that key features of PMS activities are that they should be proactive, dynamic, and responsive, so that they can detect and provide timely data on emergent risks, a pragmatic approach to survey validation may be needed. Aspects of RWP monitoring/PMS are agile processes. There must be the ability to react to changing scenarios of device use and to data from risk processes, field issues, trend monitoring, and complaints or issues identified for competitor devices. In designing surveys for these scenarios, an overly complex or time-consuming survey validation process could jeopardize timely and reactive survey deployment. A pragmatic approach to survey design and validation is particularly appropriate to the RWP monitoring of DHAs because on-market changes are often either small or iterative/incremental.^66^ A subset of RWP activities should have a focus on providing as much live data on the real use of the device as possible, and questionnaire validity relates as much to early identification of potential issues as to absolute classification of issues. If the survey is primarily designed for screening to identify on-market issues, it must be developed to require minimal time and to be highly usable for the layperson/HCP users. For some aspects of data gathering, this may take priority over the specificity of clinical information because later surveys and clinical data gathering exercises can be used to verify signals identified in data.^66^ A broad consensus screening approach for proactive PMS, in vigilance, could be followed by specifically designed surveys to explore identified issues, in the form of PMCF.

Survey validation is not an explicit requirement of the current regulations or guidelines for PMS, but it could be considered to be compulsory as part of the general quality management system requirement for validation of processes for the development of medical devices.^34^ Where surveys are used to gather noncritical, supplementary, or confirmatory data that are not essential to safety surveillance, it may be unnecessary and impractical to validate the instrument before use. An example of this is preliminary user feedback, for example, on their preferences for aspects of the style of the application or on possible future directions of development of the product.^33^ To gather this type of information, the survey will need to include highly specific questions regarding the DHA’s interface and its manner of use. Standard validated instruments would often not address the specific tool closely enough to be used, and a specifically developed survey will generally be required.^60^^,^^70^^,^^71^

Question validation ought to follow generally accepted survey validation approaches, namely assessment of validity and reliability (Figure 3). These can be validated by resorting to different techniques such as expert review, statistical correlation and agreement analysis, or literature review. Validation should not be considered an optional activity. Under medical device regulations (eg, in the EU^11^), adherence with quality standards is critical and requires structured, recorded, and validated methods to be applied in the testing and gathering of data on device performance.^34^^,^^55^ Aspects of validation to consider are language translation validation and validation against GCP/ISO 14155. In addition, the survey/tool should be validated against General Data Protection Regulation requirements.

## Summary

DHAs are becoming increasingly important to health care and greater clarity and greater innovation in regulatory thinking is required to ensure safety and performance without stifling innovation. Some take the view that the regulatory approval processes for DHA must be discrete assessment exercises on the basis of submissions of changes.^2^^,^^24^^,^^72^ Processes for PCCP on the basis of market adaptivity, with performance tracked by PCCP have been proposed in the United States, United Kingdom, and even to an early degree in proposed legislation for the EU.^18^^,^^22^^,^^23^^,^^73^ There is insufficient guidance on how to manage appropriate RWP monitoring in the context of constantly adapting DHA. DHAs are uniquely positioned to survey their users, given the interface often being on their mobile phones, and different instruments can be used to extract useful information to inform device safety and performance and meet PMS requirements.^18^^,^^22^^,^^23^^,^^73^ Methods of web-based controlled experiments and clinical development will need to move more closely together to ensure that DHAs are appropriately assessed. Surveys are a recognized regulatory method to generate data; however, the detail regarding their design, validation, and delineation with CIs requires further development. In the use of RWP-monitoring approaches, there should be clear justification that the methodology is appropriate to the specific task for which it is used, that bias in the design of instruments is avoided, and that the quality of data is understood.

## Potential Competing Interests

Dr Gilbert has or has had consulting relationships with Una Health GmbH, Lindus Health Ltd, FLO Ltd, Thymia Ltd, and Ada Health GmbH and holds share options in Ada Health GmbH. Dr Pimenta is an employee of Ada Health GmbH. Dr Stratton-Powell is an employee of RQM+. Dr Welzel declares no competing interests. Dr Melvin is an unpaid advisory board member of Pumpinheart Ltd; previously, a senior medical officer in medical devices at the Health Products Regulatory Authority, Ireland; and a previous co-chair of the Clinical Investigation and Evaluation Working Group of the European Commission.
